# Ingestion of subthreshold doses of environmental toxins induces ascending Parkinsonism in the rat

**DOI:** 10.1038/s41531-018-0066-0

**Published:** 2018-09-27

**Authors:** L. Anselmi, C. Bove, F. H. Coleman, K. Le, M. P. Subramanian, K. Venkiteswaran, T. Subramanian, R. A. Travagli

**Affiliations:** 1 Department of Neural and Behavioral Sciences, Penn State—College of Medicine, Hershey, PA USA; 2 Department of Neurology, Penn State—College of Medicine, Hershey, PA USA

## Abstract

Increasing evidence suggests that environmental neurotoxicants or misfolded α-synuclein generated by such neurotoxicants are transported from the gastrointestinal tract to the central nervous system via the vagus nerve, triggering degeneration of dopaminergic neurons in the substantia nigra pars compacta (SNpc) and causing Parkinson’s disease (PD). We tested the hypothesis that gastric co-administration of subthreshold doses of lectins and paraquat can recreate the pathology and behavioral manifestations of PD in rats. A solution containing paraquat + lectin was administered daily for 7 days via gastric gavage, followed by testing for Parkinsonian behavior and gastric dysmotility. At the end of the experiment, brainstem and midbrain tissues were analyzed for the presence of misfolded α-synuclein and neuronal loss in the SNpc and in the dorsal motor nucleus of the vagus (DMV). Misfolded α-synuclein was found in DMV and SNpc neurons. A significant decrease in tyrosine hydroxylase positive dopaminergic neurons was noted in the SNpc, conversely there was no apparent loss of cholinergic neurons of the DMV. Nigrovagally-evoked gastric motility was impaired in treated rats prior to the onset of parkinsonism, the motor deficits of which were improved by l-dopa treatment. Vagotomy prevented the development of parkinsonian symptoms and constrained the appearance of misfolded α-synuclein to myenteric neurons. These data demonstrate that co-administration of subthreshold doses of paraquat and lectin induces progressive, l-dopa-responsive parkinsonism that is preceded by gastric dysmotility. This novel preclinical model of environmentally triggered PD provides functional support for Braak’s staging hypothesis of idiopathic PD.

## Environmental triggers: a gut reaction

Tube-feeding rats with low levels of the herbicide paraquat and the dietary factor lectin triggers the development of Parkinson’s disease (PD), supporting the idea that the disease can start in the gut. Two weeks after co-administration of paraquat and lectin for seven days, Alberto Travagli and colleagues at Penn State College of Medicine, Pennsylvania, found misfolded α-synuclein in both the vagal neurocircuitry, which connects the gastrointestinal tract to the central nervous system, and the substantia nigra pars compacta where there was also a loss of dopaminergic neurons. Furthermore, the rats exhibited typical PD symptoms, including reduced gastric motility and impaired motor function, that could be alleviated with l-dopa. Removing part of the vagus nerve prevented the development of disease suggesting that environmentally triggered PD spreads from the gut.

## Introduction

While the etiology of Parkinson’s disease (PD) is unknown, both genetic and environmental factors have been theorized to play a role in its pathogenesis. In the search for environmental triggers for the development of idiopathic PD, Braak’s group hypothesized that an ingested “unknown pathogen” enters the gastrointestinal (GI) tract, and is itself either transported retrogradely via the vagus nerve to the dorsal motor nucleus of the vagus (DMV) within the brainstem, or induces retrogradely spreading neural dysfunction.^1^ As a consequence of DMV involvement, the finely-tuned vagal modulation of GI motility is disrupted.^2^ Autonomic dysfunction, including delayed gastric emptying and reduced gastric motility, can occur long before the onset of the classical motor symptoms of PD.^3–5^ The recent discovery of an anatomical connection between the DMV and the substantia nigra pars compacta (SNpc) (i.e., the nigro–vagal pathway), as well its demonstrated importance in the control of gastric tone and motility,^6^ might be the pathway by which the “unknown pathogen” travels or induces the impairment of the neurocircuit from the DMV to SNpc. Disruption of this nigro-vagal circuit may, therefore, explain the prodromal gastric dysmotility observed in PD patients.

In many studies, different pesticides, e.g., rotenone, herbicides, e.g., paraquat, and toxins, e.g., MPTP and 6-OHDA, have been administered by various routes, including oral administration, in order to model idiopathic PD.^7–12^ Although very useful as animal models, environmentally induced idiopathic PD in humans is unlikely to result from either single or multiple exposures to high doses of an agent over a short period of time. Rather, individuals are exposed to a myriad of environmental toxins over the course of their lifetime. A more likely scenario, therefore, involves repeated exposures to low doses of toxins, or a combination of toxins, whose pathogenicity may be enhanced by external factors, including diet.^13,14^ Epidemiological studies, for example, have demonstrated that the drinking of well water, as well as long-term exposure to pesticides/herbicides and heavy metals, are all associated with an increased incidence of idiopathic PD.^14–19^

Paraquat is a widely used herbicide and, given the strong positive correlation between its use and the incidence of idiopathic PD (reviewed in ref. 20), paraquat administration (up to 100 mg/kg p.o. or i.p., either alone or in combination with the fungicide, maleb), once or twice a week for three–six consecutive weeks is used commonly to induce experimental idiopathic PD.^10^ In this model, parkinsonian symptoms are observed typically after at least 4 weeks.^10,21^ Other investigators have raised issues, however, with contradictory studies that have not consistently shown loss of dopaminergic neurons in SNpc or replicated reliable parkinsonism following oral paraquat administration, while other reports have failed to show strong evidence for paraquat contamination of food.^22–25^

Dietary factors such as lectins have been implicated in the pathogenesis of idiopathic PD-like pathology in C. elegans.^20^ Lectins are ubiquitous carbohydrate-binding proteins that are present worldwide in the human diet.^26^ Lectins can penetrate the GI tract, either by endocytosis, via a breakdown in gut barrier function, or via a lectin receptor (saccharide)-mediated mechanism, and can be transported retrogradely within neurons.^27–30^ While lectins are environmentally pervasive, dietary lectins in properly cooked food are harmless and generally thought to pose no health risk.^26^ The consumption of raw uncooked vegetables, grains, and eggs that are rich in lectins, however, can potentially enhance the toxicity of pesticides and herbicides resulting in higher prevalence of idiopathic PD.^17^ By virtue of their membrane permeability, lectins have been developed as a chaperones for drugs, but have also been shown to transport viruses and toxin(s),^31,32^ including those that may be responsible for α-synuclein inclusions in PD.^33^ As such, a lectin-mediated insult is likely to be gradual, and may be influenced by association with other macro/micronutrients or ingested chemicals. It is possible, therefore, that dietary lectins contribute to the transport from the GI tract to the central nervous system (CNS) of pathogens that induce degeneration of dopaminergic neurons and Lewy body-like protein aggregation, i.e., the histological hallmark of idiopathic PD.^34^ Thus, lectins may represent a key environmental factor in the development of this disease.

In the present study, therefore, we tested the hypothesis that gastric administration of subthreshold doses of paraquat and lectins induces an ascending pattern of α-synuclein aggregation in the vagus nerve and DMV and consequent gastric dysmotility, followed by degeneration of SNpc neurons and motor features of idiopathic PD.

## Results

### Incubation of α-synuclein with subthreshold concentrations of lectin and paraquat accelerates the rate of fibril formation in vitro

**Fig. 1:**
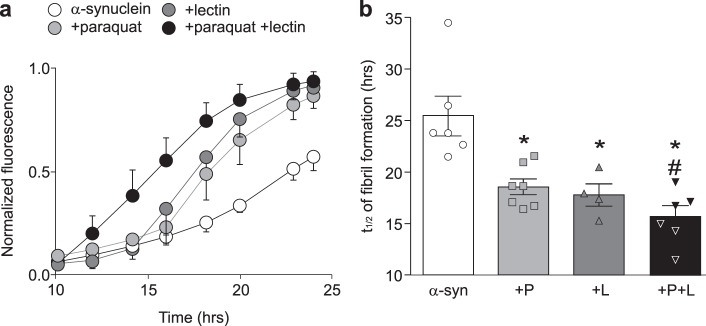
Incubation with paraquat + lectin increases the rate of α-synuclein fibrillation. **a** Time course of α-synuclein fibrillation in the presence of α-synuclein alone (white, α-syn, *n* = 6), paraquat (light gray, P, *n* = 7), lectin (dark gray, L, *n* = 4) or a combination of paraquat + lectin (black, P + L, *n* = 6). **b** Graphic summary of fibrillation *t*_1/2_ for α-synuclein. ^*^p < 0.05 vs. α-synuclein alone; ^#^*p* < 0.05 vs. paraquat or lectin

In vitro incubation of α-synuclein alone (*n* = 6) induced fibril aggregation with a half-time (*t*_1/2_) of 25 ± 1.9 h. Incubation of α-synuclein in the presence of either paraquat (100 µM; *n* = 7) or lectin (0.0025% w/v; *n* = 4) accelerated the rate of fibrillation (*t*_1/2_ = 19 ± 0.8 and 18 ± 1.1 h for paraquat and lectin, respectively, *p* < 0.05 vs. α-synuclein alone). The *t*_1/2_ fibrillation was accelerated further upon incubation of α-synuclein with a solution containing both paraquat and lectin (*t*_1/2_ = 16 ± 1.1 h, *p* < 0.05 vs. paraquat or lectin alone; *n* = 6; Fig. 1).

These data suggest that, in combination, paraquat and lectin act co-operatively to accelerate the rate of α-synuclein fibrillation in vitro, compared to either paraquat or lectin alone.

### Misfolded α-synuclein is present in myenteric neurons of the GI tract, in the DMV, and in SNpc of paraquat + lectin treated animals

**Fig. 2:**
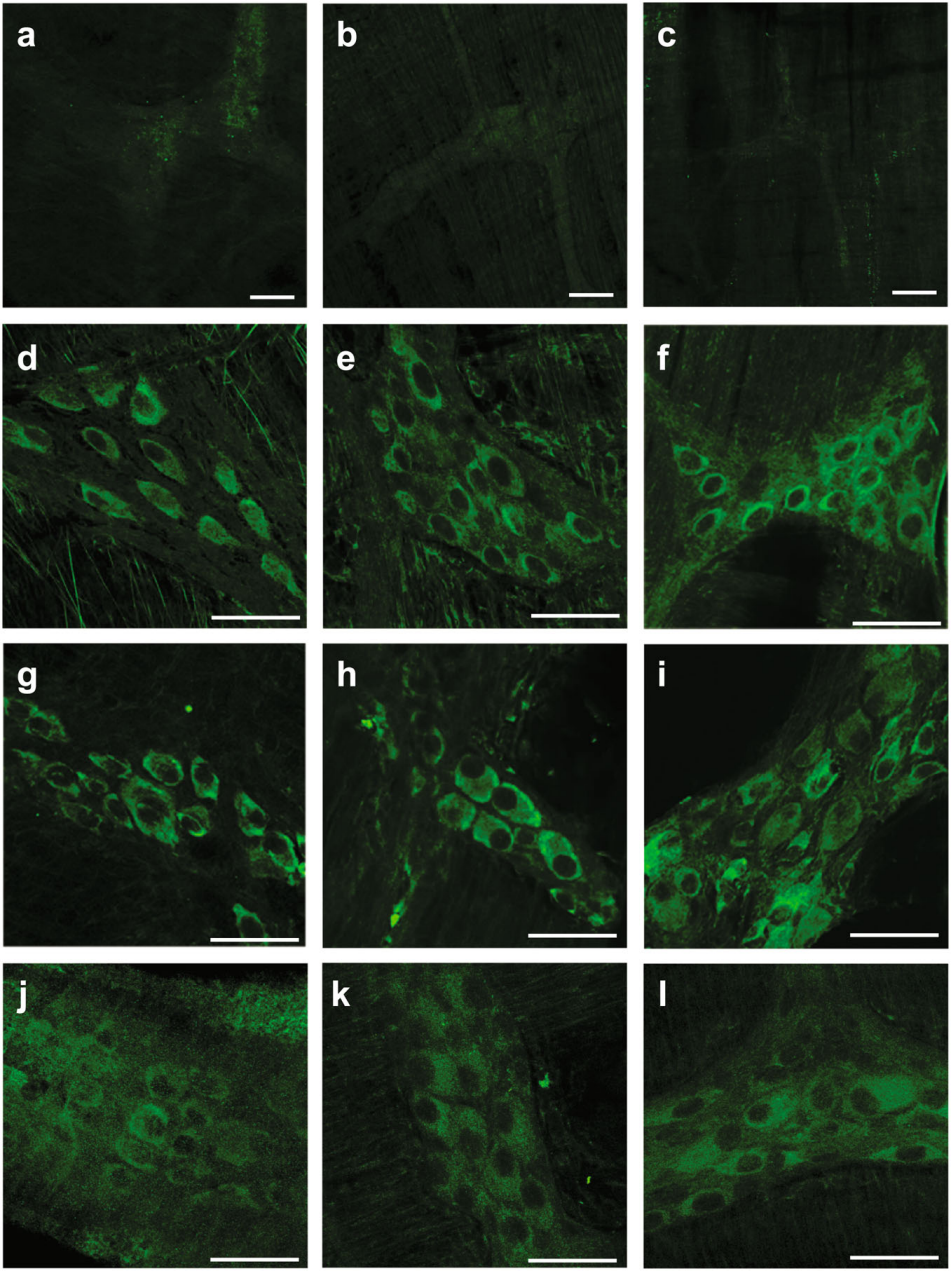
Paraquat + lectin treatment promotes α-synuclein misfolding in myenteric neurons of the GI tract. Representative micrographs showing ^129^Ser α-synuclein in the myenteric plexus isolated from the stomach **a**, **d**, **g**, **j**, the small intestine **b**, **e**, **h**, **k**, and the large intestine **c, f, i, l** from control animals (top row), animals sacrificed two (second row) or four (third row) weeks after the end of the gavage with paraquat + lectin, or animals that received subdiaphragmatic vagotomy prior to the paraquat + lectin treatment (bottom row). Calibration bars: 50 μm

**Fig. 3:**
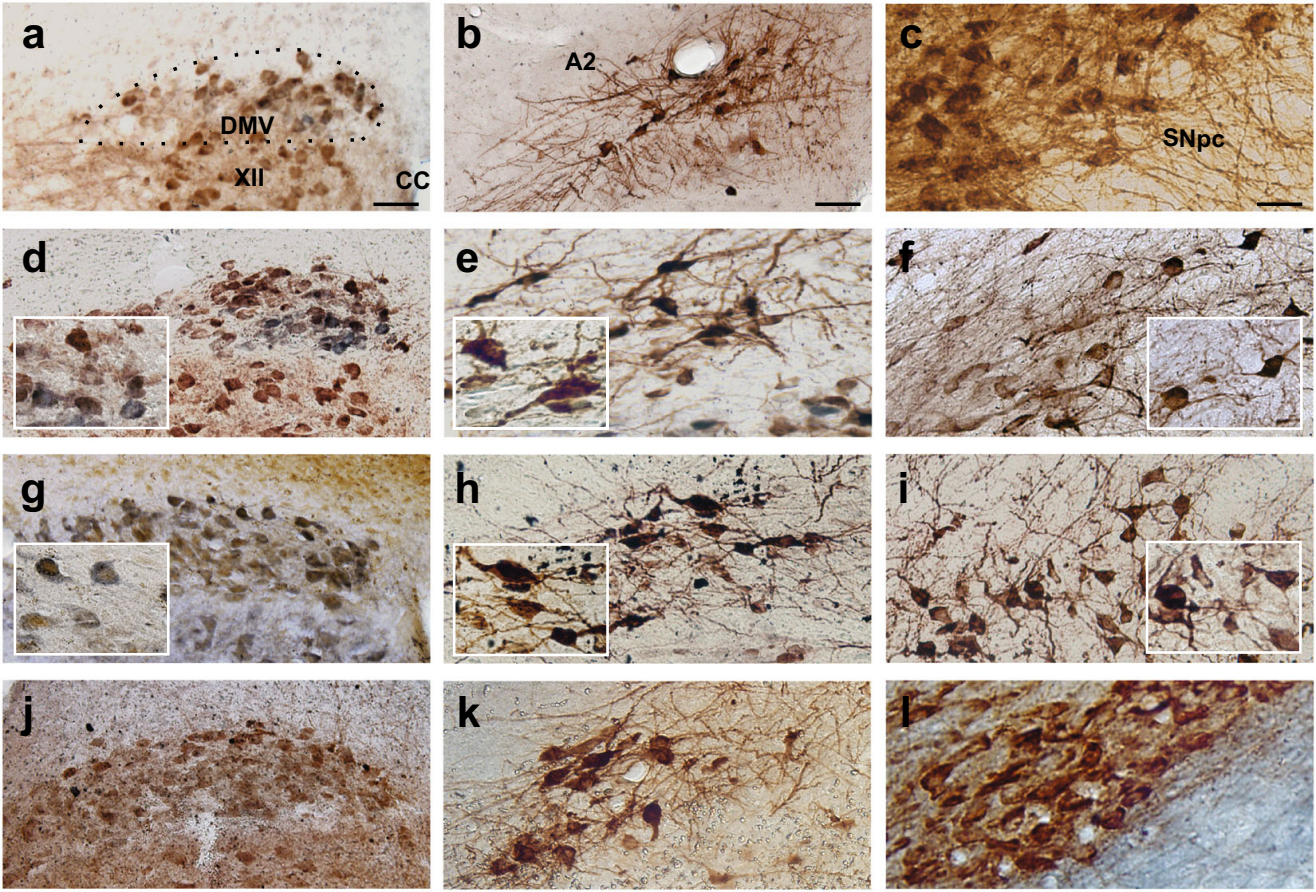
Misfolded α-synuclein is present in the DMV, the A2 area, and the SNpc after treatment with paraquat and lectin. Representative micrographs showing the co-localization of ChAT- or TH-immunoreactivity and ^129^Ser α-synuclein-immunoreactivity. Representative micrographs showing the same rostro-caudal level of the DMV in control **a**, 2 **d**, and 4 **g** weeks after the last gavage with paraquat and lectin, or in animals that received subdiaphragmatic vagotomy prior to the treatment **j**. ChAT-immunoreactivity (brown, **a**, **d**, and **j**; blue in **g**) and ^129^Ser α -synuclein (blue in **a**, **d**, and **j**; brown in **g**); calibration bar: 75 µm. Representative micrographs showing the A2 area in control **b**, 2 **e**, and 4 **h** weeks after the last gavage with paraquat + lectin, or in animals that received subdiaphragmatic vagotomy prior to the treatment **k**. TH-IR (brown) and ^129^Ser α -synuclein (blue); calibration bar: 75 µm. Representative micrographs showing the same area of the SNpc in control **c**, 2 **f**, and 4 **i** weeks after the last gavage with paraquat + lectin, or in animals that received subdiaphragmatic vagotomy prior to the treatment **l**. TH-IR (brown) and ^129^Ser α -synuclein (blue); calibration bar: 50 µm. Insets are higher magnifications of their respective panels

In control rats, ^129^Ser α-synuclein-immunoreactivity (-IR) was not detected in myenteric neurons of the stomach (Fig. 2a), small (Fig. 2b) or large intestine (Fig. 2c). Two to 4 weeks after the end of the treatment with paraquat + lectin, however, expression of ^129^Ser α-synuclein-IR was observed in myenteric neurons through the whole extent of the GI tract, including rats that received vagotomy prior to the treatment (Fig. 2d-l). At either 2–4 weeks after the end of the treatment, ^129^Ser α-synuclein-IR was also observed in choline acetyltransferase (ChAT)- and tyrosine hydroxylase (TH)-positive neurons of the DMV, the A2 area, and the SNpc (Fig. 3d-i) of treated rats, but not in control animals (Fig. 3a-c) or in animals treated with lectin of paraquat alone. In animals (*n* = 9) that underwent complete subdiaphragmatic vagotomy prior to paraquat + lectin administration, ^129^Ser α-synuclein-IR was not observed in in vagal neurons of the dorsal vagal complex (DVC) or in SNpc (Fig. 3j-l).

These results provide further support to the observation that a histological hallmark of idiopathic PD is observed in enteric neurons, as well as in key CNS nuclei following treatment with paraquat + lectin, but following subdiaphragmatic vagotomy, ^129^Ser α-synuclein-immunoreactivity is limited to myenteric neurons.

### Paraquat and lectin treated animals have impaired motor functions

**Fig. 4:**
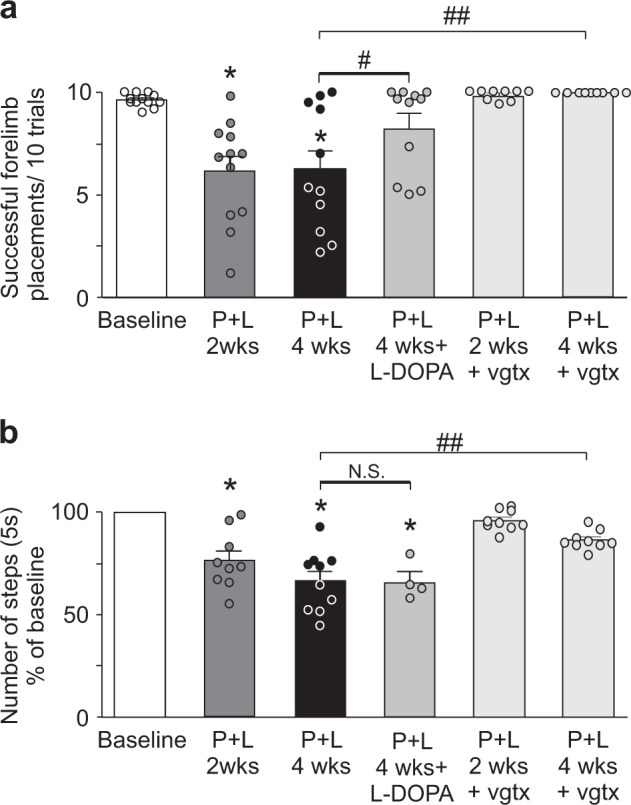
Paraquat and lectin treatment impairs motor activity. Graphic summary showing the motor performance of rats examined with the vibrissae **a** and stepping **b** tests following treatment with paraquat + lectin. Note that the motor activity was significantly reduced two weeks after the end of treatment and persisted thereafter. l-dopa pretreatment induced a significant improvement of motor performance assessed with the vibrissae test (*n* = 12). Animals that received subdiaphragmatic vagotomy (*n* = 9) prior to the treatment did not show any motor impairment. ^*^p < 0.05 vs. baseline

Prior to treatment with paraquat + lectin, the baseline score for the vibrissae test was 9.6 ± 0.1 successful forelimb placement/10 trials (*n* = 12). Two weeks after the end of the treatment, the score decreased significantly to 6.1 ± 0.7 successful forelimb placement/10 trials (*n* = 12; *p* < 0.05). The impaired motor performance showed no further deterioration, with the score 4 weeks after the end of the treatment being 6.2 ± 0.9 successful forelimb placement/10 trials (*n* = 11; *p* < 0.05 vs. baseline). A significant amelioration of Parkinsonism was evident after four doses of l-dopa, with an increase in vibrissae test scores to 8.3 ± 0.65 successful forelimb placement/10 trials (*p* < 0.05 vs. paraquat + lectin; *p* > 0.05 vs. baseline), supporting the hypothesis that exposure to subthreshold doses of paraquat + lectin induces on-going nigrostriatal dopaminergic degeneration that is reversibly ameliorated with l-dopa treatment (Fig. 4a).

A similar, but lesser impairment in motor behavior was observed with the stepping test. The baseline stepping test score was decreased significantly to 76.4 ± 4.7% (*n* = 9; *p* < 0.05 vs. baseline), 2 weeks after the end of the treatment with paraquat + lectin. The motor performance continued to deteriorate by 4 weeks after the end of the treatment to 66.4 ± 4.7% (*p* < 0.05 vs. own baseline). Administration of l-dopa did not show a significant amelioration of the stepping impairment, likely determined by the minor impairment observed in this partial forced motor test (Fig. 4b).

As expected, from the extent of the toxin-induced bilateral lesion of the nigrostriatal pathways (see below), these rats did not exhibit any rotational behavior either spontaneously or following l-dopa administration. These brief treatments did not induce any drug-related dyskinesias.

In contrast, the motor performances remained at baseline levels when rats were gavaged with lectin or paraquat alone (10 ± 0 vibrissae test, and 92.2 ± 4 and 85 ± 3% stepping test, *n* = 5 for both groups; *p* > 0.05 vs. P + L).

Similarly, rats that underwent subdiaphragmatic vagotomy prior to the paraquat + lectin treatment did not show motor impairment two weeks after the end of the gavage in either tests (vibrissae: 9.8 ± 0.1, and stepping: 96 ± 1.7% of baseline; *n* = 9; *p* > 0.05 vs. own baseline). At 4 weeks, their motor score was significantly higher than that of nonvagotomized rats at the same time point (vibrissae: 10 ± 0, and stepping: 86.6 ± 1.6% of baseline; *n* = 9; *p* < 0.05 vs. nonvagotomized rats at 4 weeks; Fig. 4).

These data indicate that paraquat + lectin treatment induces parkinsonism that is relieved by l-dopa treatment and it is prevented by subdiaphragmatic vagotomy prior to the treatment.

### Paraquat and lectin-treated animals have a decreased number of TH-positive neurons in the SNpc

**Fig. 5:**
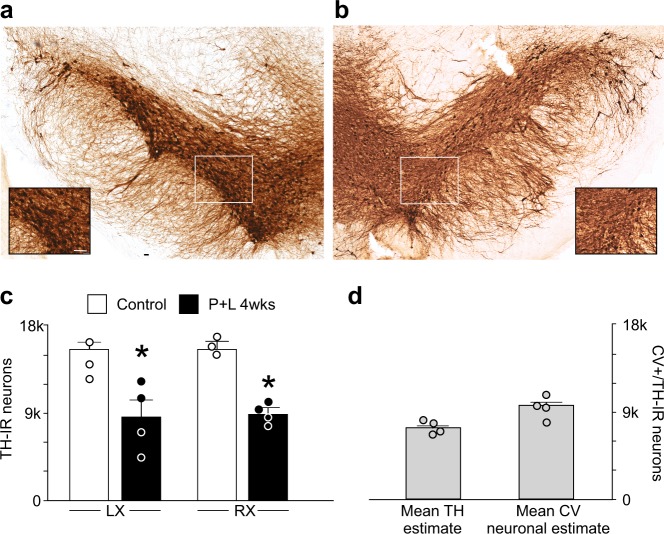
Treatment with paraquat + lectin induces loss of TH-positive neurons in the SNpc. Representative images of TH-positive neurons in SNpc of control **a** and treated animals **b**. Insets are higher magnifications of the boxed areas in the respective panels. Calibration bar: 100 µm. Graphic summary of TH-IR neuronal number in both the left **c** and right **d** SNpc of control (white) and P + L treated (black) rats. Note that a significant loss of neurons was detected in SNpc of animals 4 weeks after the last gavage of paraquat + lectin (*n* = 3 for controls and 4 for P + L 4 weeks, respectively; ^*^p < 0.05 vs. control). **d** Graphic summary showing that the mean estimate number of TH-IR neurons is not significantly different from the mean estimate number of CV-positive neurons in the SNpc of P + L treated animals (*n* = 4)

Stereological estimates showed a significant loss of TH-positive neurons in the SNpc four weeks after the end of the treatment (*n* = 3, 4 for control and P + L, respectively; *p* < 0.05). Nissl staining showed a comparable decline in SNpc neuronal number, suggesting a loss of neurons, rather than a temporary down regulation of TH expression (Fig. 5). Conversely, the number of ChAT-IR neurons in the DMV was unchanged, i.e., 1900 ± 368 and 2123 ± 126 neurons in control and paraquat + lectin treated rats, respectively (*p* > 0.05). Rats treated with paraquat or lectin alone did not show any decline in SNpc neuronal number (*n* = 5 for both groups; *p* > 0.05 vs. P + L).

These data indicate that animals treated with subthreshold doses of paraquat + lectin induces a significant, bilateral nigrostriatal degeneration of dopaminergic neurons of SNpc, but not of cholinergic DMV neurons.

### The Nigro–Vagal pathway that controls gastric motility is impaired following treatment with paraquat and lectin

**Fig. 6:**
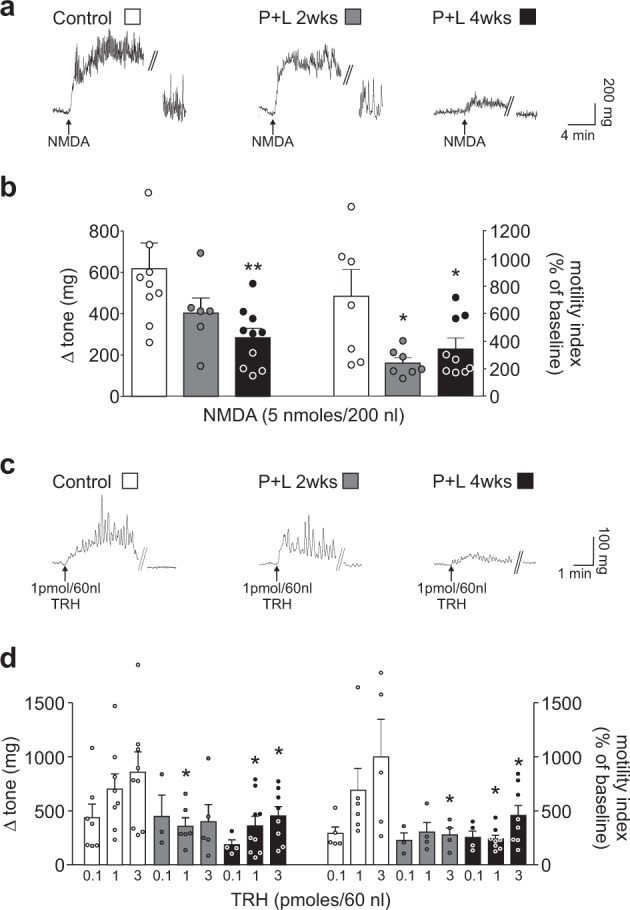
Treatment with paraquat and lectin induces impairment of the nigro–vagal pathway. **a** Representative recordings from the gastric antrum showing that the increase of tone and motility following microinjection of NMDA in the left SNpc was reduced progressively at 2 and 4 weeks after the last gavage treatment. **b** Bottom panel: graphic summaries showing that 2 or 4 weeks after the paraquat and lectin treatment, microinjection of NMDA in the left SNpc increased gastric antrum tone (*n* = 9, 6, 10 for control, P + L 2 weeks and P + L 4 weeks, respectively) and motility (*n* = 7, 7, 9 for control, P + L 2 weeks and P + L 4 weeks, respectively) to a significantly lesser extent than in controls (^*^p < 0.05 vs. control). **c** Representative recordings from the gastric antrum showing that the increase of tone and motility following microinjection of TRH (1 pmole/60 nl) in the DMV was reduced progressively at 2 and 4 weeks after the last gavage treatment. **d** Graphic summaries showing that, 2 or 4 weeks after the paraquat and lectin treatment, microinjection of TRH (0.1–3 pmoles/60 nl) in the DMV increased gastric antrum tone and motility to a significantly lesser extent than in controls (^*^p < 0.05 vs. control)

We confirmed our previous findings^6,35^ showing an increase in gastric tone and motility following microinjection of N-methyl-d-aspartate (NMDA, 5 nmoles/200 nl) into the SNpc of control rats. This NMDA-induced gastroexcitation, observed in both the antrum and corpus, was diminished markedly in rats tested either 2 or 4 weeks after treatment with paraquat + lectin. Data for paraquat + lectin are summarized in Fig. 6 (antrum) and Table 1 (corpus).

**Table 1:** Tone and motility response in gastric corpus following NMDA and TRH microinjections indicate a compromised nigrovagal innervation

	NMDA 5 nmol/200 nl in SNpc		TRH 0.1 pmol/60 nl in DMV		TRH 1 pmol/60 nl in DMV		TRH 3 pmol/60 nl in DMV	
	TONE (mg)	MOTILITY (% of baseline)	TONE (mg)	MOTILITY (% of baseline)	TONE (mg)	MOTILITY (% of baseline)	TONE (mg)	MOTILITY (% of baseline)
Control	410 ± 51.9	553 ± 135	593 ± 133	369 ± 120.2	419 ± 81.7	803 ± 139.7	439 ± 77.5	1020 ± 351.9
P + L 2 weeks	211 ± 63.8^*^	307 ± 49.7	136 ± 91.5^*^	247 ± 75	776 ± 272	270 ± 20^*^	433 ± 228	326 ± 90.9
P + L 4 weeks	255 ± 37^*^	289 ± 38.2^*^	161 ± 42.3^*^	229 ± 61.9	353 ± 75.9	332 ± 66.7^*^	522 ± 121	536 ± 98.8

^*^p < 0.05 vs. control.

Values expressed as mean ± S.E.M.

Similarly, we confirmed previous reports^36^ showing that DVC microinjection of thyrotropin-releasing hormone (TRH, 0.1–3 pmoles/60 nl) increased gastric tone and motility in control rats in a dose-dependent manner. Following paraquat + lectin treatment, however, the TRH-induced increase in tone and motility was reduced significantly. Data are summarized in Fig. 6 (antrum) and Table 1 (corpus).

Treatment with either paraquat (*n* = 5) or lectin (*n* = 5) alone, did not alter significantly any of the gastric responses to NMDA or TRH microinjection.

To evaluate if the attenuated increase in gastric tone and motility observed in treated animals in response to central microinjection of either NMDA or TRH was due to an impairment of the gastric smooth muscle functionality, bethanecol was administered (dose: 10 µg/kg i.v.). The bethanecol-induced increases in gastric tone and motility were comparable among all groups, indicating that paraquat + lectin treatment did not compromise gastric smooth muscle. Data are summarized in Table 2.

**Table 2:** The gastric motility response to bethanecol i.v. administration shows no alteration of smooth muscle functionality

	Bethanecol i.v. (10 µg/kg)		Bethanecol i.v. (10 µg/kg)	
	Antrum		Corpus	
	Tone (mg)	Motility (% of baseline)	Tone (mg)	Motility (% of baseline)
Control	142 ± 85.0	214 ± 47.6	138 ± 79.4	210 ± 5.0
P + L 4 weeks	166 ± 49.14	160 ± 18.4	161 ± 40.8	194 ± 22.5

Values expressed as mean ± S.E.M.; *p* > 0.05

Together, these data support the hypothesis that gastric administration of subthreshold doses of paraquat + lectin induces an impairment of the nigro–vagal pathway without compromising gastric smooth muscle.

## Discussion

In the present study, we demonstrated that co-administration of subthreshold doses of lectin + paraquat produce (i) consistent pathological hallmarks of α-synuclein aggregation in enteric, brainstem, and midbrain neurons, (ii) stable parkinsonism associated with modest, but significant, degeneration of SNpc dopaminergic neurons, and (iii) motor parkinsonism is reversibly treatable with l-dopa. We also demonstrated a sequential progression of α-synuclein aggregation, with inclusions in the DMV preceding those in the SNpc. This temporal pattern of central dysfunction was mirrored functionally, with dysregulated gastric responses to stimulation of either the nigro–vagal pathway or the DVC preceding the development of motor parkinsonism. Finally, animals that received subdiaphragmatic vagotomy prior to paraquat + lectin administration did not show motor parkinsonism, and the accumulation of misfolded α-synuclein was confined to myenteric neurons, further supporting a vagally mediated progression of the synucleopathy.

The temporal pattern and progression of parkinsonism described in our current animal model replicates faithfully the predictions of a temporally distinct pattern for idiopathic PD^1,37,38^ as outlined by Braak’s staging hypothesis, i.e., a spread of synucleopathy that starts with the ingestion of an unknown pathogen, which enters myenteric neurons of the enteric nervous system, then travels to the CNS via retrograde transport through the vagus nerve, affecting the DMV (without causing neuronal loss) and the fine vagal modulation of GI motility first and, later, higher areas including the dopaminergic neurons of the SNpc, thus impairing motor functions.

Herein, we have shown that subdiaphragmatic vagotomy restricted misfolded α-synuclein to gastric myenteric neurons following paraquat + lectin administration, with no progressive synucleinopathy being observed in either DMV or SNpc, or Parkinsonism, thus supporting a ENS–vagal route of the pathology. Support for the involvement of the vagus nerve in different phases of PD is found in studies showing that patients who received truncal vagotomy, thereby severing the myenteric neuron–vagal–DMV connection, showed a clear reduction in the incidence of PD.^39^ Moreover, a potential vagal pathology in PD is reinforced by findings that the electrogastromyography patterns of PD patients are similar to those of vagotomized patients.^40,41^

Despite multiple studies demonstrating that misfolded α-synuclein can spread and propagate in a prion-like fashion, it is important to note that it is likely that other factors such as unique histological features, mitochondrial stress, and cytosolic calcium levels are responsible for the regional distribution of idiopathic PD pathology.^42^ Therefore, it is possible that a combination of the spread of pathogenic α-synuclein together with endogenous factors renders neurons susceptible to damage.

As with most paradigms, the experimental models of PD used currently have many advantages and disadvantages.^43,44^ A plethora of studies have shown that exposure to paraquat is correlated positively with parkinsonism in humans.^14,45,46^ Indeed, several studies have reported that systemic chronic administration of high doses of paraquat in experimental animals induce some of the hallmark parkinsonian disturbances.^13,14,23,25,45,47^ The ability of paraquat itself to cause idiopathic PD has, however, been called into question due to the lack of reproducibility of the pathology using oral dosing models, the high doses used in most animal studies, the lack of paraquat residues in food, and scientific fraud in some reported studies (reviewed in recent letter by Cook et al.^22^).^7–11,24,48^ Other toxin models that have stable behavioral outcomes, such as the 6-OHDA induced model in rats, require intracranial administration of the toxin, while systemically administered toxins like rotenone, MPTP, and paraquat do not replicate the route of entry of environmental toxins.^10,49^ Moreover, these models induce severe Parkinsonism over a relatively short-time period, which does not reflect the temporal pattern and progressive nature of idiopathic PD. Additionally, models that overexpress α-synuclein, either via genetic manipulation or via recombinant vector administration,^50^ either do not replicate the natural course of the disease or fail to replicate the full repertoire of motor deficits.^43^

In contrast, our model replicates the likely route of environmental agents that instigate idiopathic PD pathology, namely ingestion and enteric entry, follows a temporally defined pattern inducing brainstem disruption that precedes the nigral pathology that results in bradykinesia, i.e., the behavioral hallmark of idiopathic PD. The extent of the behavioral deficits observed in our model are milder and more variable, which also simulates the natural course of early idiopathic PD. Our data also provide a putative reason for the variability noted in previous oral paraquat toxicity models in that co-administration of lectin is required to induce a more consistent pathology. Furthermore, this raises the possibility that consumption of raw lectin-rich food in rural communities where chronic environmental exposure to toxins such as paraquat is endemic, provides an underlying mechanistic basis for the pathogenesis of idiopathic PD.^13–18,47^ Ingestion of such a diet, therefore, by virtue of the chemical properties of lectins^27–30^ may facilitate the absorption and/or transport of toxins in susceptible individuals.^31,32^ At the doses and administration route, i.e., oral gavage, used in the present study, however, lectins or paraquat, when administered alone, did not induce any significant effect on the parameters analyzed, i.e., motor performance, gastric emptying, tone and motility, and immunohistochemical properties.

Our data suggest that impairment of both the recently described nigro-vagal,^6^ as well as the TRH-activated vagal efferent pathway^36^ occur in the absence of functional disruption of the gastric smooth muscle itself. Such gastric dysfunctions were observed prior to impairment of motor control and in advance of SNpc neuronal degeneration, mimicking the prodromal GI issues observed in many parkinsonian patients.

There has also been considerable debate on the mechanism(s) through which paraquat may enter the CNS across the blood–brain barrier.^51^ Results from our study suggest that the presence of lectins is also required to induce α-synuclein aggregation in the gut as well as its spread into the CNS via the vagus nerve, and subsequently into the SNpc via the newly discovered nigro–vagal pathway.^6^ Further support for this novel mechanism of action is provided by the recent description of α-synuclein accumulation in a subpopulation of enteroendocrine cells that exhibit neuron-like properties and have direct connections to enteric and/or extrinsic nerves.^52^ Indeed, the suggestion has been raised that α-synuclein itself could act as a lectin.

In conclusion, our study shows that the ability of orally administered subthreshold doses of paraquat in the presence of lectins to trigger parkinsonian pathology. Although we did not characterize the mechanistic features of this model, the range of neurodegenerative and pathophysiological changes induced by co-administration of paraquat + lectin reproduces many of the cardinal features of the human disease, including, parkinsonism that is responsive to l-dopa therapy, neurocircuit dysfunction, induction of neuronal α-synucleinopathy, neurodegeneration leading to the loss of SNpc dopaminergic neurons while sparing the DMV, as well as prodromal gastric motility disturbances that were observed in absence of smooth muscle impairment. The appearance of gastric dysfunction and Parkinsonism, its prevention by subdiaphragmatic vagotomy, and the distinct sequence of pathological and degenerative changes described herein, make for an attractive experimental model that will help identify triggering factors essential to idiopathic PD etiology, as well as being of use in the discovery of biomarkers and testing of new therapeutics at a stage where interventions would be disease-modifying rather than symptom-alleviating.

## Methods

All procedures were conducted in accordance with the National Institutes for Health guidelines, with the approval of the Penn State University-College of Medicine Institutional Animal Care and Use Committee and according to journal policies and regulations on animal experimentation.

### In vitro α-synuclein fibril formation

To examine the effect of paraquat and lectin on the kinetics of fibril formation, an in vitro fibrillation assay was used, as described previously.^53^ Briefly, solutions containing: (i) purified recombinant α-synuclein alone (35 µM in 50 mM Tris-HCl buffer, pH 7.5); (ii) lectin from *Pisum sativum* (0.0025%); (iii) paraquat (100 µM); or (iv) a combination of lectin and paraquat, were incubated at 37 °C with constant shaking at 300 rpm for ~40 h. Each sample was plated in triplicate on a 96-well plate, and 20 µM Thioflavine T, a fluorescent dye that binds to fibrillary structures, was added. The fluorescence (excitation at 450 nm and emission at 485 nm) was measured at different time points using a fluorescence plate reader (Spectramax Gemini EM, Molecular Devices, Sunnyvale, CA) interfaced with Softmax® pro 6.3.1 software (Molecular Devices). The relative fluorescence units were averaged and plotted as a function of time; the resulting plot was interpolated, normalized and fitted to a sigmoidal curve using GraphPad Prism® software (GraphPad Software, LaJolla, CA, USA).

### Animals and treatment

Male Sprague–Dawley rats were housed in an AAALAC accredited Animal Care Facility maintained at 24 °C on a 12:12 h light/dark cycle. Food and water were provided ad libitum. Rats were gavaged daily, for seven consecutive days, with 1% sucrose (control; *n* = 12) or (i) 1% sucrose and 0.05% lectin from *P. sativum* + paraquat (1 mg/kg, P + L; *n* = 20), (ii) 1% sucrose and 0.05% lectin (L; *n* = 5), or (iii) 1% sucrose and paraquat (1 mg/kg, P; *n* = 5). To promote absorption, gastric emptying was delayed by injection of cholecystokinin (3 µg/kg i.p.) 15 min prior to each gavage. Rats were allowed to recover 2 (*n* = 9) or 4 (*n* = 11) weeks before experimental procedures were carried out. A group of rats received injections of l-dopa (4 mg/kg) and benserazide (15 mg/kg, i.p. diluted in ascorbate saline; *n* = 11) twice a day for 2 days, after the third week of recovery. Rats treated with lectins at doses up to 0.2% were observed for up to 12 weeks.

A group of rats was anesthetized with isoflurane (2.5% in 100% O_2_) and an abdominal laparotomy was performed to expose and severe both posterior and anterior subdiaphragmatic vagal branches, as described previously.^6,54^ The efficacy of the vagotomy was assessed with i.p. administration of 0.2 mg/kg fluorogold.

### Tissue collection

At the conclusion of the behavioral or gastric experiments (see below), rats were euthanized under deep general anesthesia, rapid sternal thoracotomy and transcardiac perfusion with 200 ml of heparinized saline followed by 200 ml of 4% paraformaldehyde (PFA) in PBS. Brains were removed and postfixed in 4% PFA and 20% sucrose for 24–48 h at 4 °C, and then transferred in a solution containing PBS, 0.08% Na azide, and sucrose. The brains were sliced in 50μm-thick coronal sections using a freezing microtome using either a 1:4 or 1:8 systematic random sampling routine and preserved as floating sections prior to further processing.

### Immunohistochemistry

Detailed methodology has been described previously.^55,56^ Primary antibodies were (i) rabbit-α-^129^Ser α-synuclein (Abcam, Cambridge, UK; 1:1000); (ii) goat-α-ChAT (Chemicon, Temecula, CA; 1:5000); (iii) mouse-α-TH (Immunostar, Hudson, WI; 1:10000) or rabbit α-TH (Pel-Freez biological, Rogers, AR; 1:200). For immunoperoxidase staining, secondary antibodies were biotinylated donkey immunoglobulins (IgGs) for multiple labeling (Jackson ImmunoResearch Laboratories, West Grove PA) diluted 1:1000; the detection complex was ExtrAvidin-horseradish peroxidase (ExtrAvidin-HRP; 1:1500). For immunofluorescence staining, secondary antibodies were donkey immunoglobulins Alexa Fluor 488 or 568 (ThermoScientific, Waltham,MA; 1:1000).

Both primary and secondary antibodies were incubated at room temperature on a shaker for 3 days or overnight, respectively. Brain slices were rinsed in PBS, mounted on gelatin-coated slides, air-dried overnight, dehydrated in alcohol, cleared in xylene, coverslipped with DePeX (Electron Microscopy Sciences, Hatfield, PA, USA).

### Behavioral testing

A well-established rodent behavioral battery of tests^57,58^ was used to identify the parkinsonian phenotype in treated rats, as described previously.^59^ Briefly, these consist of the (1) vibrissae-evoked forelimb placement test (*Vibrissae test*), a forced reflex test in which the tester restrains three limbs and allows stimulation of the ipsilateral vibrissae to evoke a reflex ipsilateral forelimb placement on a firm surface. This test is repeated 10 × 3 at each testing session. (2) Stepping test,^58,59^ a partial-forced reflex test in which the experimenter holds the testing rat, restraining both hind limbs and one forelimb at a time, with the free forelimb touching a flat surface. The rat is moved sideways along the surface at a rate of 90 cm/5 s in the direction of the testing forelimb. The test is repeated 3× separately for both forelimbs. Results are expressed as percentage of baseline. (3) Post l-dopa/benserazide treatments twice daily for 3 days and behavioral assessment using the vibrissae test at 1 and 2 hours post-treatment.

These motor behavioral tests were used to assess the parkinsonian phenotype prior to treatment (baseline), every week thereafter, as well as on the day of the gastric motility test. To avoid any pharmacological interaction, the last l-dopa treatment was conducted at least 1 week prior to the gastric motility studies

### Stereology

In each animal, an entire series of brain sections (1:4 or 1:8), containing the whole SNpc or the whole DVC, were stained using cresyl violet (CV) to identify key anatomical structures and structural integrity. Brain slices in representative groups were stained for TH as described below. TH-positive neurons in the SNpc were quantified using the Stereo Investigator software suite from MBF bioscience with a 100x magnification using a Olympus BX53 microscope (Olympus, Tokyo, Japan) fitted with a digital CCD camera (Hamamatsu, Hamamatsu City, Japan) and a motorized stage (Prior Scientific, Rockland, MA, USA). The total numbers of cells were estimated using the optical fractionator,^60^ the coefficient of error was calculated according to Gundersen et al.,^61^ and values ≤0.05 were accepted as significant. TH stained sections were counterstained with CV to assess neuronal loss, as opposed to TH down-regulation, and estimated independently using design based stereology as detailed above.

### Gastric studies

Gastric tone and motility recordings were performed as described previously.^6,54^ Briefly, animals were fasted overnight (water ad libitum) before being anaesthetized deeply with Na-thiobutabarbital (Inactin;® 100–150 mg/kg i.p). After intubation with a tracheal catheter, a midline laparotomy was performed and two custom-made 6 × 8 mm strain gauges (AT Engineering, Hershey, PA) were sutured to the serosal surface of the anterior gastric corpus and antrum in alignment with the circular smooth muscle. Leads were exteriorized, prior to suturing the abdominal laparotomy; the jugular vein was catheterized to permit systemic administration of bethanechol (10 µg/kg), a muscarinic agonist that does not cross the blood-brain barrier and excites the smooth muscle directly supramaximally. Animals were then placed in a stereotaxic frame and were instrumented for measuring the effects of microinjections in SNpc and DVC on gastric tone and motility as described previously.^6^

The ionotropic glutamate receptor agonist, NMDA, (5 nmoles/200 nl) was microinjected into the SNpc (in mm, rostro-caudal (RC): −5.0 to 5.6 from bregma; medio-lateral (ML): 1.6–2.4 from midline; dorso-ventral (DV): −7.6 to 7.8 from the surface of the dura mater). To assess the effects of direct activation of vagal efferent motoneurons, TRH (0.1–3 pmol/60 nl) was microinjected into the left DVC (in mm, RC: 0.0–0.6 from calamus scriptorius; ML: 0.2–0.4 from midline; DV: 0.5–0.65 from the brainstem surface). All drugs were dissolved in isotonic phosphate buffered saline (PBS, in mM: 115 NaCl, 75 Na_2_HPO_4_, 7.5 KH_2_PO_4_; pH = 7.4).

Strain gauges signals were acquired with a Wheatstone bridge, filtered (low pass filter cutoff = 0.5 Hz; AT Engineering), amplified (EXP CLSG-2; QuantaMetrics, Newton, PA, USA) and recorded on a computer using Axotape® 10 software (Molecular Devices, San Jose, CA). Gastric tone and motility were recorded for 2–5 min before and 15–20 min after drug application; the drug-induced effects on tone and motility were calculated through average value of the calibration measures as described previously.^6,35^ Since variations in size of the animal and in the strain gauge placement may lead to slight differences in responses between individual animals, each animal served as its own control, and motility data were measured as percentage changes over baseline (=100%).

Gastric motility was calculated using the following formula, as described previously:^6^

$${\mathrm{Motility}}\,{\mathrm{index}}\,{\mathrm{percent}} = \left[ {\left( {{N}1 \times 1} \right) + \left( {{N}2 \times 2} \right) + \left( {{N}3 \times 4} \right) + \left( {{N}4 \times 8} \right)/{t}} \right] \times 100.$$

Where *N* = number of peaks in a particular force range and *t* = interval time in which the gastric motility is measured. *N*1 = 20–59 mg, *N*2 = 60–100 mg, *N*3 = 101–200 mg, *N*4 ≥ 201 mg.

### Materials

Unless indicated otherwise, all chemicals were obtained from Sigma-Aldrich (St. Louis, MO).

### Statistical analysis

Data are reported as mean ± SEM and in all instances significance was set at *p* < 0.05.

Data were evaluated using one-way ANOVA followed by post-hoc Tukey’s multiple comparison test or one sided, paired *t*-test using GraphPad® software (Graph Pad Prism).

## Data Availability

The data are maintained by Dr. R. Alberto Travagli, department of Neural and Behavioral Sciences Penn State College of Medicine, and can be made available for review upon request.
